# Cerebro-Spinal Meningitis

**Published:** 1863-07

**Authors:** N. S. Davis

**Affiliations:** Professor of Clinical Medicine, &c.


### CEREBRO-SPINAL MENINGITIS.

By N. S. DAVIS, M.D.

We cannot better answer numerous letters that we have received, inquiring in relation to the best mode of treating a disease which has prevailed very severly in some localities in the North-West, during the past year, than by publishing the following observations. The disease has been called by different names; and it is quite certain that different diseases have been sometimes confounded together. In a recent number of the *Boston Medical and Surgical Journal*, we find eight cases, with *post mortem* examinations, reported by Dr. UPHAM, under the head of "Congestive Fever" or "Cerebro-Spinal Meningitis." At the conclusion of his cases, he sums up the symptoms as follows:—

"In its *mode of attack*, the disease was commonly sudden and without premonition, the patient, for the most part, continuing on duty and making no complaints till the very day of his seizure. Some of the most violent cases thus commenced; Case XII, previously cited, is, in point, where the soldier appeared with his company at the evening dress parade, complained of chilliness, headache, &c., during the night, and was dead within thirty-six hours following. And the subjects of the disease, in most cases, were those previously in the fulness of robust health,—between the ages of 18 and 24,—who had endured hardships and exposures with impunity.

"The *symptoms* were, at the first, headache, referred oftentimes to the back part of the head particularly, with dizziness,—pain in the back and limbs, this last, occasionally, of an excruciating character,—with, sometimes, rigors, and nausea, and vomiting. Chilliness, rather a well-defined chill, characterized the accession of the disease. A peculiar stiffness in the muscles of the face and neck was often an early symptom; this would be followed by local spasms, perversion of vision, &c. In some cases, the initiatory symptoms were those of a severe cold, with a disposition to paralysis of the tongue and a portion of the muscles of the face. With this the respiration would be difficult and irregular, giving occasion to fear a congestive attack of the lungs. There was often tenderness at the nape of the neck and along the spine early in the disease. The skin was, usually, moist, but hot. The face was suffused,—of a dusky hue,—and the features distorted in the manner before mentioned,—the eyes congested and suffused. There was not, for the most part, active delirium,—but perversion of intelligence rather, and dulness and indifference to outward objects, from which condition the patient could be roused and made to answer questions consciously. The tongue had, at first, a white creamy coat, which, in the course of the disease, became yellowish or brown at centre and base, more rarely dry and cracked towards the close. There was loss of appetite, but, usually, not very urgent thirst. The heart's action was irregular, sometimes tumultuous, to which the pulse did not always respond, being mostly accelerated, but not strong,—occasionally intermittent. The bowels were regular, or inclined to diarrhœa and costiveness by turns. Petechiæ were not an unfrequent manifestation,—in appearance almost identical with the true typhus eruption, and, like that, seen upon every part of the body, except the face,—persistent on pressure, varying in hue from the darkest aspect of the measles to that of the true petechial spots imbedded in the skin. Purpural spots, abundant and of large size, were sometimes present, and were always a grave symptom. There was no marked tenderness of the epigastrium or abdomen. In the cases of longer duration, there was, in the last stages, sordes on the teeth and lips, and involuntary evacuations of urine and fæces. The patients often die without much symptoms of ex-

20

haustion. The decubitus was mainly on the side, with the head not unfrequently thrown back,—the neck rigid and stiff, —a partial opisthotonos. There was, uniformly, great restlessness and jactitation. As an accompaniment and occasionally a sequel to the disease, iritis was several times observed. So, also, was synovitis,—and, in one instance, pericarditis. The above are among the more prominent and constant symptoms, —but there was a considerable diversity in the manifestations of the disease during its progress, whether towards a favorable or fatal result; in no one case do I remember to have seen even a majority of those I have enumerated present.

"Singular and anomalous symptoms were sometimes noticed. Dr. Jewett, Surgeon of the 51st Mass. Regt., to whom I am indebted for a clear and able account of the disease, as it occurred in the troops under his care, reports that 'in a single case, a pleasing delirium was noticed, with loquacity and decidedly erotic desires, accompanied with priapism more or less extensive during the greater part of the disease.' This peculiarity, he adds, was noticed in about one-third of his cases. Dr. Cowgill alludes to the same fact. Dr. Jewett noticed the decubitus upon the dorsum among fourteen cases which occurred in the 51st Mass. Regt. in but a single instance. 'In all the others,' he observes, 'the patients lay upon the side till near the close of life.' 'In a few cases, and those the most severe ones,' he also remarks, 'no moan or sound of any kind escaped the patient, but there was a fearful restlessness, which ceased only at death; in others, there was much moaning.' Stiffness of the muscles of the face, before alluded to, amounting at times to spasm, was almost pathognomonic. In some form, this affection was present in nearly all the cases sent in by Dr. Ware; it was common in those treated in Academy Hospital. Dr. Jewett speaks of it as being present in fully one-third of the cases which came under his observation, 'there being,' as he says, 'more or less stiffness of the muscles of the neck and back, with opisthotonos,—in one case, paralysis of the glosso-pharyngeal nerve, and, in two others, eversion of the eyes and occasional squinting.'

"The *duration* of the affection varied from a period of less than thirty-six hours, to that of three, four, or six weeks, and even longer. According to my own observation, the more usual duration has been from three or four to seven days."

From the foregoing detail of symptoms, given by one of the most accurate observers of the present time, it is evident that the cases of disease called "Cerebro-Spinal Meningitis," vary much in their symptoms, rapidity of their progress, and in their results. It would seem that the most constant and characteristic symptoms are, sudden and severe pain in the head and back; great restlessness, especially in paroxysms; either mental depression or delirium; more or less rigidity of the muscles of the neck, and often of the back and extremities; and a hurried and variable condition of both circulation and respiration. The disease is often so rapid that it terminates fatally during the second or third day after the attack, while, in a few instances, it lingers several weeks. The *post mortem* appearances are almost as variable as the symptoms during life. In a majority of cases, a sero-purulent fluid is found in the ventricles and under the base of the brain, with a white crudy or membraneous exudation on the surface of the pia mater, constituting evidence of a rapidly suppurative inflammation; but, in other cases, no trace of disease is visible in any part of the brain or its membranes. Thus, in one case related by Dr. UPHAM, the only important pathological condition present on the *post mortem* was the existence of six or eight ounces of a sero-purulent fluid in the pericardium. In other cases, the lungs suffer most, and are often found extensively congested or hepatized. When the disease prevails, epidemically, its course is, generally, more rapid and fatal than almost any other disease with which the practitioner has to contend. We were informed by a medical friend, a few weeks since, that in one locality in the southern part of Indiana, an epidemic had prevailed during the past spring, of such severity that more than sixty deaths took place in the practice of a single physician within a few weeks. The more prominent symptoms, as described to me, were, sudden and severe prostration, with fever, soreness and stiffness in the fauces and muscles of the neck; pain in the head, neck, and chest; suffusion of the eyes; frequency of pulse and respiration; more or less muscular rigidity; delirium; petechial or purpuric spots on the surface; and death, generally, in from two to six days.

It will be remembered that, about twenty years since, a very fatal epidemic prevailed in many parts of the Middle and Western States. It was styled the "Black Tongue," in the newspapers. As it appeared in the interior of New York, the great majority of cases presented all the characteristics of a malignant erysipelas,—attacking, first, the throat and extending rapidly over the face, head, neck, and sometimes a large part of the surface of the trunk of the body; but, in numerous instances, instead of developing external erysipelatous inflammation, the soreness of the fauces was quickly followed by delirium; muscular twitchings or rigidity, especially in the muscles of the neck; frequent pulse; hurried and irregular breathing; restlessness; great prostration; and death. In Michigan and other highly-malarious districts, the latter form of the disease was the most prevalent. In all these cases, where *post mortem* examinations were made, sero-purulent effusions, membranous exudations, and the indications of inflammation were found in the ventricles, and under the base of the brain, and on the medulla oblongata. Hence, in the localities in which this form of disease was present, it came to be recognized and described as an epidemic,—cerebro-spinal meningitis. During the prevalence of that epidemic, we were practicing in Binghamton, N. Y., and but few cases occurred in that town. During the whole fourteen years that we have resided in Chicago, no epidemic of this disease has prevailed in the city or its immediate vicinity. From the middle of March to the first of June, of this year, cases of erysipelas were of much more frequent occurrence than usual; and many of the cases were accompanied by symptoms of unusual malignancy. About the middle of June, we were called in quick succession to such a number of cases presenting symptoms of cerebro-spinal inflammation, that we became apprehensive that they formed the beginning of an epidemic prevalence of the disease. The three following cases will sufficiently illustrate both the symptoms and treatment of the disease as it has presented itself, in our practice, during the last thirty days:—

CASE I.—A boy, aged about 12 years; nervous temperament, and rather feeble constitution, was attacked with headache sufficient to cause him to leave the school-room on the 13th of June. The next morning he was better, and made a visit to some friends a few miles in the country. He returned home on the afternoon of the 15th; and, after walking from the railroad depot to his father's residence, he complained of feeling tired; and, in the evening, was attacked with violent pain in the head, neck, and back,—but most severely in the head. I saw him at 9 o'clock the same evening: I found him lying partly on his side, with a sad and anxious expression of countenance; skin dry and moderately hot; pulse 120 per minute, but soft; tongue slightly coated; and bowels quiet. He complained of severe pain in the head, with soreness of the throat and stiffness of the muscles of the neck. There were frequent muscular twitchings in the extremities, with nausea and efforts at vomiting whenever he attempted to assume the upright position. The parents thought the sickness was caused by injudiciously eating fruit in the country. They were assured, however, that the symptoms strongly indicated a serious degree of irritation in the base and central parts of the brain.

About three hours later, a severe paroxysm of general convulsions occurred, followed by increased rigidity of the muscles of the neck, constant tossing of the arms, moaning, redness of the eyes, and complete stupor, with dilitation of the pupils. Five hours later, he remained unconscious; conjunctiva injected and pupils dilated; head moderately hot; pulse 130 per minute, and weak; bowels had been freely moved; the extremities cool, and lips pale; more decided rigidity of the muscles of the neck; and slight drawing of the head to one side; and almost constant irregular action of the muscles of the extremities.

Seven hours later, the general symptoms remained the same, except the constant rigidity of the muscles of the neck had extended also to those of the shoulders and upper extremities, fixing the arms firmly against the sides of the chest, and the forearms moderately flexed; but the lower extremities were still being constantly tossed about. Six hours later, all muscular agitation or tossing had ceased, but some rigidity of the muscles of the neck and arms remained; the pulse had become extremely frequent, variable, and weak; the respiration irregular, with mucus accumulating in the air-passages; slight involuntary discharges of both urine and fœces; deglutition suspended. Respiration, circulation, and muscular action continued steadily to diminish until the patient died, about thirty-six hours from the time that his symptoms attracted serious attention. No *post mortem* examination was allowed. The treatment of the case consisted in cold applications to the head, a pillow of pounded ice to the occiput and back of the neck, a mercurial purgative, followed by moderately full doses of iodide potassa and belladonna; and, after the first eighteen hours, quinine and other remedies were prescribed; but the difficulty of deglutition had become such that very little was actually swallowed.

Case II.—I was called to see Mrs. H., a native of Ireland, aged about 36 years, the mother of several children, on the afternoon of June 17th, 1863. Found her suffering intense pain in the head and upper part of the spine; anxious expression of countenance; position dorsal, with inclination to right side; rigidity of the muscles of the neck and flexors of the hands and feet, so as to draw the thumbs into the palms of the hands and fix the fingers and toes in a state of semi-flexion; pluse 120 per minute and small; surface of the head and trunk above the natural temperature, but extremities cool; respiration hurried and somewhat irregular; tongue covered with a white moist coat; and bowels quiet. On inquiry, I learned that the patient had complained of pain in the head for two days past. Twelve hours previous to my visit, the pain in the head had become greatly increased, accompanied by a general convulsion, lasting only a few seconds, and since which time she has lain in the condition she was found on my arrival. I should not omit to state, that the convulsive paroxysm in the morning was immediately preceded by vomiting, and that nausea and efforts to vomit had occurred several times during the day, more especially when any attempt was made to change the position of the patient. I directed the head and back of the neck to be covered with a sac of pounded ice, and dry warmth to the extremities. Directed to be taken internally, calomel, 3 grs., bicarb. soda, 3 grs., every two hours until the bowels were evacuated. I also directed fifteen drops of tincture of belladonna to be given in connection with one drachm of the *sulphite* of lime every two hours. Eighteen hours after the commencement of this treatment, I found her with less pain in the head and back; expression of countenance less anxious; muscular rigidity of the extremities less, but not entirely gone; fœces and urine had both passed freely in bed, without the control of the patient; and the pupils were moderately dilated, but whether from the effects of the belladonna or the disease it was not easy to decide. The calomel and soda was discontinued; the sulphite of lime and tincture of belladonna continued in the same doses, but at intervals of once in three hours, and six grains of iodide of potassa dissolved in camphor water given half way between. The cold applications were also continued to the head and neck.

June 19th.—3 o'clock P.M., all the symptoms improved. The rigidity of the muscles of the extremities had entirely ceased; the pain in the head and the stiffness of the muscles of the neck continued slightly; pulse 90 per minute; fœces and urine passed naturally; and patient cheerful. The pupils of the eyes were dilated and the throat dry, evidently, as the effects of belladonna. The doses of sulphite of lime and belladonna were now reduced to half a drachm of the first and eight drops of the last, given every four hours, alternated with the iodide of potassa. The ice cap to the head and neck was exchanged for simple cloths wet in hydrant water. Animal broth given in small quantities, at short intervals, for nourishment.

June 20th.—The patient appears to be entirely convalescent. While lying quiet, she feels no pain, and shows no stiffness or rigidity of the muscles, though there is a general of soreness in the flesh, great weakness, and, when she moves, the head is light and giddy. Continued the sulphite of lime and belladonna at intervals of once in eight hours, but omitted all other medicines. Allowed a more liberal use of nourishment, consisting of beef-tea, milk-porridge, &c.

June 22d.—Found the patient sitting up half an hour, and convalescence fully established. Ordered twenty drops of muriated tincture of iron to be taken at each meal-time as a tonic, and omitted all other medicines. The patient has since continued well.

Case III.—June 17th, at 4 o'clock P.M., I was called urgently to 114 West Jackson Street, to see a child about two years old, reported to have "cramps." I found the child lying in a recumbent dorsal position, inclined towards the left side; the face flushed; eyes suffused; head hot, and drawn moderately to the left side by rigid contraction of the muscles of that side of the neck; respiration hurried, and sometimes moaning; pulse small, tense, and frequent; frequent muscular twitchings, and, apparently, delirium. I learned that, during the latter part of the preceding night, the child had become hot, restless, and subject to sudden startings. The restlessness and fever increased until about 9 o'clock A.M., when there was a moderate general convulsion, after which, the child remained in the same condition as I found it at 4 o'clock P.M. I prescribed the same remedies, in all respects, as in Case II., only suiting the doses to the age of the child; and the result was the same, namely, the complete recovery of the patient in about one week.

During the two weeks intervening between the 15th and 30th of June, four other cases of a similar character occurred in my practice, two adults and two children. All but one were in the western division of the city. The symptoms presented in these cases led me to regard them as genuine attacks of cerebro-spinal disease; while the rapidity with which they followed each other, and preceded by an unusual prevalance of erysipelas, caused me to seriously apprehend the commencement of an epidemic cerebro-spinal meningitis. Hence, after the speedy termination of the first case, I used, in the treatment of subsequent ones, such remedies as previous reflection had satisfied me would be most likely to prove beneficial in that disease.

From all the facts I have been able to gather concerning the symptoms, progress, and *post mortem* appearances in the epidemics of cerebro-spinal meningitis that have occurred at various periods of time and in numerous localities, I am constrained to regard the disease as an asthenic inflammation of the cerebrospinal nervous centres with their investing membranes, accompanied by a highly *septic* condition of the blood and consequent rapid failure of the vital properties throughout the whole organization, with the equally rapid formation of sero-purulent effusions at the seat of local inflammation. That such a septic condition of the whole mass of the blood actually exists, is proved by the frequent appearance of dark red and purpuric spots on different parts of the cutaneous surface, the early formation of sero-purulent effusions and infiltrations, and the rapidly fatal results. Further observations may show that it holds the same relation to erysipelas as is claimed for epidemic purpural peritonitis. If we admit the correctness of the pathology just stated, it affords a basis for three rational and well-defined therapeutic indications, viz.:—

First.—To introduce, as rapidly as possible, such remedies as will neutralize or destroy the *septic* condition of the blood.

Second.—To counteract or diminish the vascular turgescence or accumulation of blood in the cerebro-spinal centres.

Third.—To maintain the depurative processes by which the system is relieved from the presence of effete and offending matter, by increasing the activity of the excretory organs.

The experiments of Dr. POLLI, with the sulphites of lime and soda, to which allusion has been made in former numbers of this Journal, together with the experience I had acquired with the first of those salts in the treatment of malignant erysipelas, caused me to resort to the sulphite of lime as the agent most likely to efficiently counteract the septic state of the blood, and fulfil the first indication named. If one of those severe and fatal epidemics, such as has been described as prevailing in some parts of the country, and, quite recently, in the vicinity of Philadelphia, I should, not only, exhibit the remedy in full doses to those actually attacked, but should give moderate quantities to such well persons as were immediately exposed in families and houses where some were already sick, to test its prophylactic virtues. While using this or such other remedies as experience may prove to be efficacious, for the purpose of correcting the septic or faulty condition of the blood, I should endeavor to lessen the local inflammatory action by such remedies as increase the *contraction* of the cerebro-spinal capillaries, and thereby lessen the accumulation of blood in those parts. For this purpose, belladonna given internally, in such doses as will speedily induce, in a moderate degree, its specific effects on the pupils of the eyes and the fauces, aided by the constant application of pounded ice to the occiputo-cervical region, constitutes our most reliable resource; for I am fully satisfied that the views of Dr. Brown Sequard, in relation to the action of belladonna, ergot, &c., are correct; and, if so, all the preparations of opium and alcoholic liquids, by their tendency to dilate the cerebro-spinal capillaries, and thereby favor the accumulation of blood, are decidedly *contra-indicated* in the treatment of the disease under consideration. In a disease so severe and rapid in its course, we should not only endeavor to correct the morbid condition of the blood, and lessen the accumulation of blood in the parts involved in local inflammation, but the excretory functions should also be carefully maintained, for the double purpose of aiding to lessen the determination of blood to the head, and of favoring the expulsion of any effete or poisonous material that may exist in the system. For this purpose, I prefer calomel and bi-carbonate of soda, sufficient to move the bowels, to be followed by full doses of iodide potassa.

Such are my views, very briefly expressed, concerning the nature and treatment of the epidemic form of cerebro-spinal meningitis.

